# EPO regulates neuronal differentiation of adult human neural-crest derived stem cells in a sex-specific manner

**DOI:** 10.1186/s12868-023-00789-1

**Published:** 2023-03-06

**Authors:** Tarek Niemann, Johannes F.W. Greiner, Christian Kaltschmidt, Barbara Kaltschmidt

**Affiliations:** 1 Molecular Neurobiology, University of Bielefeld, Bielefeld, Germany; 2 Department of Cell Biology, University of Bielefeld, Bielefeld, Germany

**Keywords:** Sexual dimorphism, Erythropoietin, Adult neural-crest-derived stem cells, Neuronal differentiation, Axon length, Neuronal morphology and plasticity

## Abstract

**Background:**

Sexual differences in the biology of human stem cells are increasingly recognized to influence their proliferation, differentiation and maturation. Especially in neurodegenerative diseases such as Alzheimers disease (AD), Parkinson’s disease (PD) or ischemic stroke, sex is a key player for disease progression and recovery of damaged tissue. Recently, the glycoprotein hormone erythropoietin (EPO) has been implicated as a regulator of neuronal differentiation and maturation in female rats.

**Methods:**

In this study, we used adult human neural crest-derived stem cells (NCSCs) as a model system for exploring potential sex specific effects of EPO on human neuronal differentiation. We started with expression validation of the specific EPO receptor (EPOR) by performing PCR analysis in the NCSCs. Next, EPO mediated activation of nuclear factor-κB (NF-κB) via Immunocytochemistry (ICC) was performed, followed by investigating the sex-specific effects of EPO on neuronal differentiation by determining morphological changes in axonal growth and neurite formation accompanied by ICC.

**Results:**

Undifferentiated male and female NCSCs showed a ubiquitous expression of the EPO receptor (EPOR). EPO treatment resulted in a statistically profound (male p = 0.0022, female p = 0.0012) nuclear translocation of NF-κB RELA in undifferentiated NCSCs of both sexes. But after one week of neuronal differentiation, we could show a highly significant (p = 0,0079) increase of nuclear NF-κB RELA in females only. In contrast, we observed a strong decrease (p = 0,0022) of RELA activation in male neuronal progenitors. Extending the view on the role of sex during human neuronal differentiation, here we demonstrate a significant increase of axon lengths in female NCSCs-derived neurons upon EPO-treatment (+ EPO: 167,73 (SD = 41,66) µm, w/o EPO: 77,68 (SD = 18,31) µm) compared to their male counterparts (+ EPO: 68,37 (SD = 11,97) µm, w/o EPO: 70,23 (SD = 12,89) µm).

**Conclusion:**

Our present findings therefore show for the first time an EPO-driven sexual dimorphism in neuronal differentiation of human neural-crest derived stem cells and emphasize sex-specific variability as a crucial parameter in stem cell biology and for treating neurodegenerative diseases.

**Supplementary Information:**

The online version contains supplementary material available at 10.1186/s12868-023-00789-1.

## Background

Erythropoietin (EPO) is a glycoprotein growth factor with an approximate molecular weight of 30.4 kDa firstly described in 1953 by Allan J Erslev as a specific kidney hormone stimulating the production of red blood cells [1, 2]. Therefore, patients with anemia-related diseases like chronic kidney disease, viral infections and leukemia, are commonly treated with EPO [3, 4]. Bunn and colleagues showed that regulation of EPO is based on low oxygen level (hypoxia) promoting the expression of hypoxia inducible factors (HIFs) and leading to increased production and secretion of EPO [5]. The secreted EPO travels via plasma to the bone marrow and stimulates viability, proliferation, and terminal differentiation of erythroid precursors via interaction with its specific cell type surface receptor (EPOR), leading to an increase in erythrocyte mass supporting the oxygen transfer to the target tissue [6, 7,8].

Besides its role in mediating erythroid progenitor maturation and therefore regulating the oxygen transport efficiency, EPO-mediated signal transduction is reported to be involved in several other biological systems like the liver, heart, vasculature and the central nervous system [5, 9, 10]. In the central nervous system (CNS), EPOR is abundantly expressed during brain development in mammals such as humans, monkeys and mice [11]. In this line, Tsai and coworkers demonstrated that knockdown of EPOR-expression leads to reduced neuronal cell proliferation in the mice brain and also reduced neurogenesis after a stroke [12]. Furthermore, expression of erythroid-specific EPOR after total knockdown showed an rescuing effect resulting in survival and enhanced neuronal cell proliferation and viability [5, 13, 14]. In this regard, EPO has also been shown to have beneficial effects on neurons, including the regulation of neuronal anti-apoptotic factors, reduction of inflammation and reduction of ROS stress [15–17].

On molecular level, binding of EPO to EPOR leads to phosphorylation of Janus tyrosine kinase-2 (JAK-2) resulting in the dissociation of nuclear factor-κB (NF-κB) from its inhibitor domain (IκB) and translocation into the nucleus [15]. The transcription factor NF-κB is known to be involved in the regulation of a broad range of biological processes such as proliferation, immune response, neuroprotection and differentiation [18–20]. Here, the activation of the individual subunits plays a crucial role, as high subunit specificity has already been demonstrated in various processes [19, 21]. In particular, the REL subunit seems to play a crucial role in the development and homeostasis of the adult mammalian brain, as its activity has already been directly linked to synaptic plasticity, neuroprotection, function and transmission [21–25]. Here we used human neural crest-derived stem cells, which are optimally suited for analyzing neuronal differentiation [26–29].

In the different behavior of stem cells, the sex of the individuals is increasingly recognized as a crucial parameter [30]. In this regard, Marin-Husstege and colleagues were able to show a interaction between male and female sex hormones and the proliferation and maturation of oligodendrocyte progenitors [31]. Hillerer and coworkers also demonstrated the circulation of sex hormones to regulate hippocampal neurogenesis [32]. Such sex-specific differences can also be seen in the human system. During glutamatergic differentiation of human neural crest-derived stem cells, we previously demonstrated that neurons derived from female NCSCs reveal a significantly increased RELA mediated neuroprotection against H_2_O_2_ induced apoptosis [33]. Nevertheless, the exact molecular interaction of EPO and the role of sex during neurogenesis remains ambiguous. Research into EPO mediated effects initially in vitro could be an important step in unraveling these questions.

## Methods

### Cell culture

Human nasal inferior turbinate stem cells (ITSCs) were obtained via minimally invasive surgical procedure according to Hauser and colleagues taking into account of local and international guidelines [26]. All experimental procedures were ethically approved by the ethics board of the medical faculty of the University of Münster (No. 2012–015-f-S). Experiments were performed using ITSCs from 5 male and 6 female donors depicted in the specific experimental figures. Cells were cultivated in TC25 flasks (Sarstedt, Nürnbrecht, Germany) containing preheated (37 °C) stem cell medium consisting of DMEM F12 mixed with 0.1 mg/ml penicillin/streptomycin (Sigma Aldrich, Taufkirchen, Germany), 200 mM L-glutamine (Sigma Aldrich), 20ng/ml FGF2 (Peprotech, Rocky Hill, USA), 20 ng/ml EGF (Peprotech), 6% B-27™ supplement (50X) (Thermofisher Scientific, Waltham, USA) with additional 10% human blood plasma (BP) (Institute for Laboratory- and Transfusion- Medicine, Heart- and Diabetes-Centre NRW, Bad Oeyenhausen, Germany) in a humidified incubator (Binder, Tuttlingen, Germany) with hypoxic atmosphere containing 5% O_2_ and 5% CO_2_ at 37 °C. The cells were fed every 2 days with fresh stem cell medium, until the matrix was digested completely. For passaging, the medium was removed and adherent cells were washed with 1x Phosphate-buffered saline (PBS) (Sigma Aldrich). After removal of PBS, 3ml of collagenase solution containing 40 mg collagenase powder type A (Worthington Biochemical Corporation, Lakewood New Jersey, USA), 49 ml PBS and 50 µl of 3 M calcium chloride (labmade) was transferred to the flasks for 20–30 min until the cells were detached for further processes.

### Pharmacological treatment of ITSCs

After standard cultivation described above, 5 × 10^4^ Cells were seeded on roughened cover glasses and cultured until all cells were completely adherent. Subsequently, 0.3, 0.5, 1, 2.5, 5 and 10 U/ml EPO (Sigma Aldrich) was added to the cells followed by incubation for 30 min at 37 °C for proliferation assays. For immunocytochemical stainings with NF-κB subunit specific antibodies only 0.5, 1, 2.5, 5 and 10 U/ml EPO was added to the cells followed by incubation for 30 min at 37 °C. For neuronal differentiation 1 U/ml EPO was used due to the promising results of the previous experiments.

### Neuronal differentiation

For neuronal differentiation, cells of 3 male and 3 female donors were cultivated in DMEM high glucose (Sigma Aldrich) containing 200 mM L-glutamine and 10% fetal calf serum (Sigma Aldrich) at a density of 5 × 10^4^ cells/well in a 12-well plate on top of roughened cover glasses. After 2 days, 1 µM dexamethasone (Sigma Aldrich), 2 µM insulin (Sigma Aldrich), 500 µM 3-isobutyl-1-methylxanthine (Sigma Aldrich), 200 M indomethacin (Sigma Aldrich) and 200 µM ethanol were added to the medium to induce neuronal differentiation (neuronal induction medium, NIM). In addition, the test group was supplemented with 1 U/ml EPO during neuronal differentiation. Medium exchange and EPO treatment was performed every 2–3 days. 9 days later, cells were additionally induced adding 0.5 µM retinoic acid (Sigma Aldrich) and 1x N-2 supplement (Gibco, Darmstadt, Germany). Afterwards, half of the medium was removed and replaced with pre-warmed NIM containing 1x N-2 supplement. Neuronal differentiation of ITSCs was performed for 1, 3, 6 and 10 weeks.

### Camera lucida tracing

For determining neuronal morphology and calculating axon length and neurite counts, images of neuronal differentiated ITSCs were analyzed in CorelDRAW 2021 version 23.1.0.389 (Core Corporation, Ottawa, Canada). The outline of the cell body was traced and the neuron was transferred to an adjusted Sholl ring, where the axonal length can be measured.

### RT-PCR

PCR was performed using Taq DNA Polymerase with TermoPol® Buffer (New England Biolabs, F.a.M, Germany) according to manufacturers guidelines with the following primers for EPO-R (GAA GTA GTG CTC CTA GAC GCC, CCT CGT AGC GGA TGT GAG AC) and GAPDH (CAT GAG AAG TAT GAC AAC AGC CT, AGT CCT TCC ACG ATA CCA AAG T).

### Immunocytochemistry

ITSCs were seeded on roughened cover glasses with a density of 5 × 10^4^ Cells. Immunocytochemistry was done as previously described in Ruiz-Perera et al. [33]. Afterwards, Primary antibodies against RELA (mouse, 200,301,065, 1:5000, Rockland, PA. USA), RELB (rabbit, D7D7W, 1:500, Cell Signaling Technology, Danvers, USA), c-Rel (rabbit, 4727, 1:500, Cell Signaling Technology), vGlut-II (rabbit, 07-1402-I, 1:300, Merck Millipore, Burlington, USA), NF200 (mouse, SAB3200747, 1:200, Sigma Aldrich), MAP2 (mouse, sC-390,543, 1:500, Santa Cruz, Dallas, USA) and synaptophysin (rabbit, ab32127, 1:500, Abcam, Cambridge, UK) were incubated for 1 h at RT. Secondary fluorochrome-conjugated antibodies (Alexa 488 anti-rabbit, Alexa 555 anti-mouse, Alexa 555 anti-rabbit, 1:300, Life Technologies, Germany) were incubated for 1 h at RT under the exclusion of light, followed by DAPI nuclear counterstaining. Fluorescence microscopy was performed using the confocal laser scanning microscope LSM 780 (Carl Zeiss, Jena, Germany). Randomly placed pictures were analyzed using ImageJ [34]. Nuclear fluorescence intensity was determined using the “measure” function in ImageJ on the nuclei of the cells.

### Statistical analysis

Data were evaluated with at least 3 biological replicates and were statistically verified with the Software Prism V8.4.3 (GraphPad Software, Inc., San Diego, CA, USA) using the Mann-Whitney test. Significance value of p < 0.05 was considered as statistically significant. The presented data shows the means ± standard deviation (SD).

## Results

### Erythropoietin treatment results in NF-κB subunit RELA nuclear translocation in adult human neural crest-derived stem cells

**Fig. 1:**
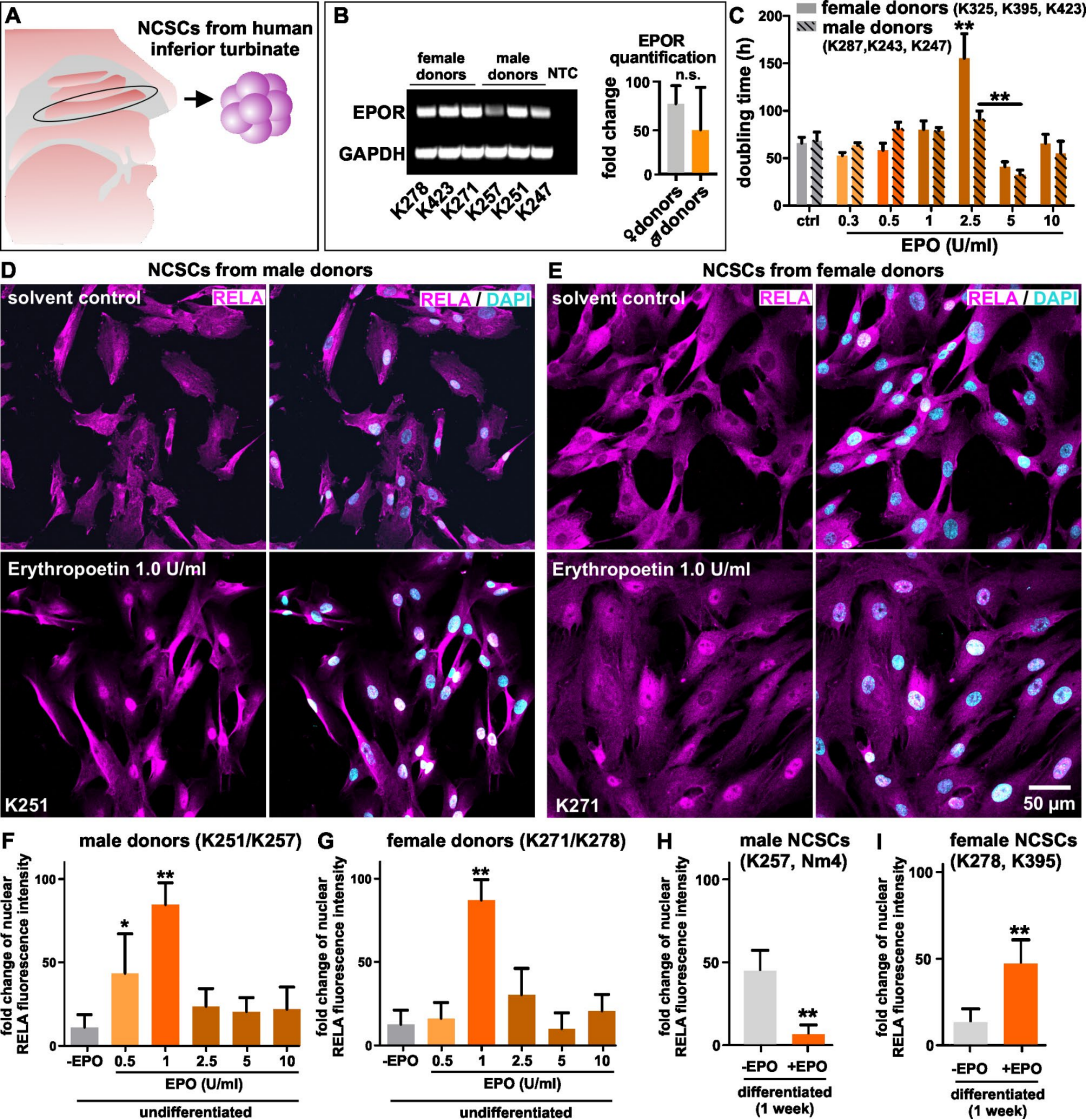
**Erythropoietin activates NF-κB RELA in human NCSCs.** A: NCSCs were derived from the inferior turbinate of the human nasal cavity. B: NCSCs derived from both sexes express human Erythropoietin receptor (EPO-R). EPO-R and GAPDH bands were cropped on top of each other. C: 2.5 U/ml EPO supplementation during cultivation significantly increased the doubling time of female NCSCs. Additionally, 5 U/ml EPO reduced the doubling time of male NCSCs compared to 2,5 U/ml EPO treatment. D, E: Immunocytochemistry revealed a strong nuclear translocation of NF-κB after 30 min of 1 U/ml EPO exposure in male and female NCSCs. F: Fold change of nuclear RELA fluorescence intensity is significantly increased in NCSCs derived from male donors after treatment for 30 min with 0.5 and 1 U/ml EPO. G: Fold change of nuclear RELA fluorescence intensity is significantly increased in NCSCs derived from female donors treated with 1 U/ml EPO for 30 min. H: After treatment of male NCSCs with 1 U/ml EPO for one week, the differentiating cells show a strong decrease of NF-κB RELA staining. I: In contrast, female NCSCs treated with 1 U/ml EPO for one week showed a strong increase of NF-κB RELA. Mann-Whitney test, *p < 0.05, **p < 0.01, ***p < 0.001. was considered significant

To initially assess the potential of inferior turbinate stem cells isolated from the human nasal cavity (Fig. 1A) to respond to erythropoietin we performed RT-PCR on cells derived from male and female donors. Undifferentiated ITSCs showed a homogenous expression of human Erythropoietin receptor (EPO-R) in both sexes (Fig. 1B). After 5 days of cultivation and supplementation with 2.5 U/ml EPO every 2 days a significant sex specific difference was shown regarding the doubling time of the NCSCs. In addition, the doubling time of male NCSCs is significantly reduced when the cells were supplemented with 5 U/ml EPO compared to 2,5 U/ml EPO. (Fig. 1C). To investigate the effects of EPO on activation of NF-κB subunits RELA, RELB and c-REL, Immunocytochemistry of confluent growing NCSCs were performed after 30 min of 0.5, 1, 2.5, 5, 10 U/ml EPO treatment. Confocal laser scanning microscopy analysis of the cells revealed a strong nuclear translocation of RELA in both sexes after 30 min of 1 U/ml EPO exposition (Fig. 1D, E). For quantification, fold change of nuclear fluorescence intensity was calculated. After 30 min of 1 U/ml EPO supplementation, nuclear fluorescence intensity of RELA was significantly increased (male p = 0.0022, female p = 0.0012) in cells derived from male and female donors (Fig. 1F, G). Interestingly, a lower dose of 0.5 U/ml also led to a significantly increase (p = 0,0260) of the nuclear RELA, but only in male NCSCs (Fig. 1F). Moreover, EPO seems to have no impact on the nuclear translocation of NF-κB RELB (figure S1 A, C). Of note, the nuclear signal of c-REL seemed to be reduced after 1, 2.5, 5, and 10 U/ml of EPO treatment in NCSCs derived from male donors (figure S1 B). This trend was also observed in female cells again without significant validation (figure S1 D). κ We additionally differentiated NCSCs derived from male and female donors for one week and performed immunocytochemistry to mark RELA nuclear translocation during differentiation. In male NCSCs nuclear level of RELA is significantly reduced (p = 0,0022) upon 1 U/ml EPO supplementation during neuronal differentiation, compared to the negative control in Fig. 1H and figure S2 (A) On the other site, supplementing female NCSCs with 1 U/ml EPO during 1 week of neuronal differentiation resulted in a significantly increased (p = 0,0079) somatic and nuclear elevation of RELA levels as can be seen in Fig. 1I and figure S2 (B) Conclusively, we detected EPO as a strong mediator of NF-κB RELA nuclear translocation in undifferentiated NCSCS from both sexes. During neuronal differentiation of NCSCs for 1 week treated with EPO, we were able to show, that a long lasting nuclear RELA translocation only occurred in female cells, whereas in male cells RELA staining decreased significantly.

### Erythropoietin mediates enhanced neuronal differentiation in female NCSCs

**Fig. 2:**
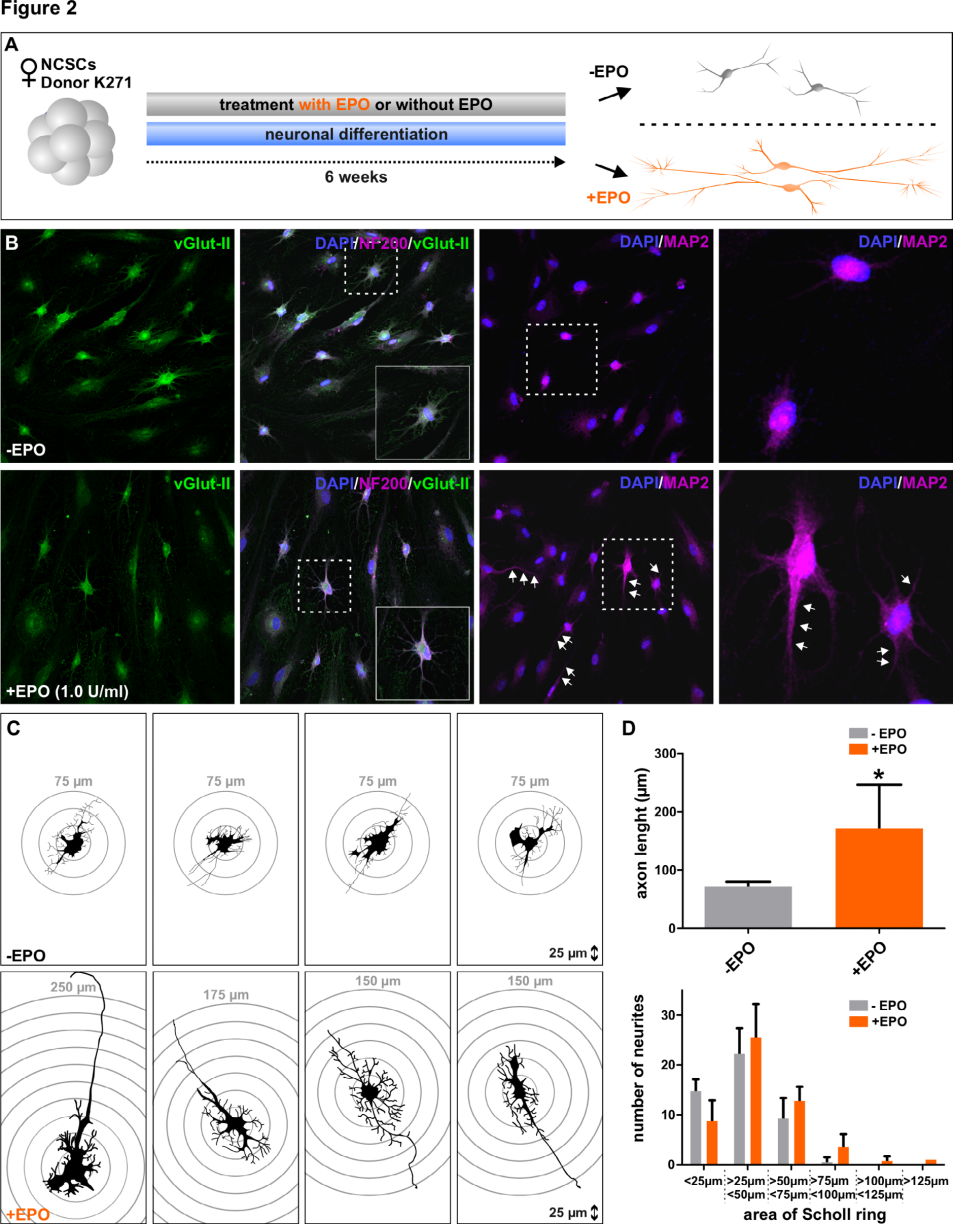
**Erythropoietin enhances neuronal differentiation in female NCSCs.** A: Schematic summary of neuronal differentiation with EPO treatment (1 U/ml) for six weeks. B: Immunocytochemistry revealed a homogenous expression of vGlut-II. On the other hand, expression of NF200 and MAP-2 is slightly increased in NCSCs derived from female donors. White arrows depict MAP2 positiv axons. C, D : Camera Lucida Tracing reveals elongated axons of female NCSCs upon EPO supplementation. (Mann-Whitney test, *p < 0.05, **p < 0.01, ***p < 0.001. was considered significant

To investigate further effects of EPO, we tested whether EPO has an impact on neuronal differentiation of neural crest-derived stem cells. For this purpose, female NCSCs underwent glutamatergic neuronal differentiation supplemented with 1 U/ml EPO for 6 weeks (Fig. 2A). The concentration of EPO during neuronal differentiation was set to 1 U/ml because this concentration was the most effective to activate NF-κB RELA (Fig. 1F, G). Immunocytochemistry of female NCSCs revealed a ubiquitous expression of glutamatergic neuron marker vesicular glutamate transporter 2 (vGlut-II) (Fig. 2B). Moreover, the expression of neurofilament-200 (NF200), a cytosomal marker of functional neurons as well as microtubule associated protein 2 (MAP2), a marker for mature terminally differentiated neurons, are slightly increased in the EPO treated group, compared to their solvent control (Fig. 2B). 37,5% of the EPO treated neurons are NF200 positive, compared to the solvent control, with only 5,3% NF200 positive cells. Moreover, 23,1% of the EPO treated neurons are MAP2 positive contrary to the untreated neurons with only 9,1% MAP2 positive cells. For axonal length determination and neurite formation the cytoskeletal stained neurons were analyzed using Camera Lucida Tracing (Fig. 2C, D). Representative female neurons revealed a change in morphology upon EPO supplementation. In particular, the axon length is significantly increased (p = 0,0286) in NCSCs treated with EPO during differentiation (Fig. 2C). The average axon length of female NCSCs after 6 weeks of neuronal differentiation without EPO treatment is 71,51 (SD = 8,69) µm, whereas the female NCSCs which has received EPO supplementation during neuronal differentiation presented an average axon length of 171,29 (SD = 75,69) µm (Fig. 2D). Accordingly, we only observed neurites above 100 μm Sholl ring range upon EPO treatment, whereas untreated ITSC-derived neurons resulted in no neurite formation longer then 75 μm (Fig. 2D). In conclusion, 6 weeks of neuronal differentiation of female NCSCs supplemented with EPO, resulted in an advanced neuronal morphology and increased mature neuronal marker expression.

## Erythropoietin regulates neuronal differentiation of neural crest-derived stem cells in a sex dependent matter

**Fig. 3:**
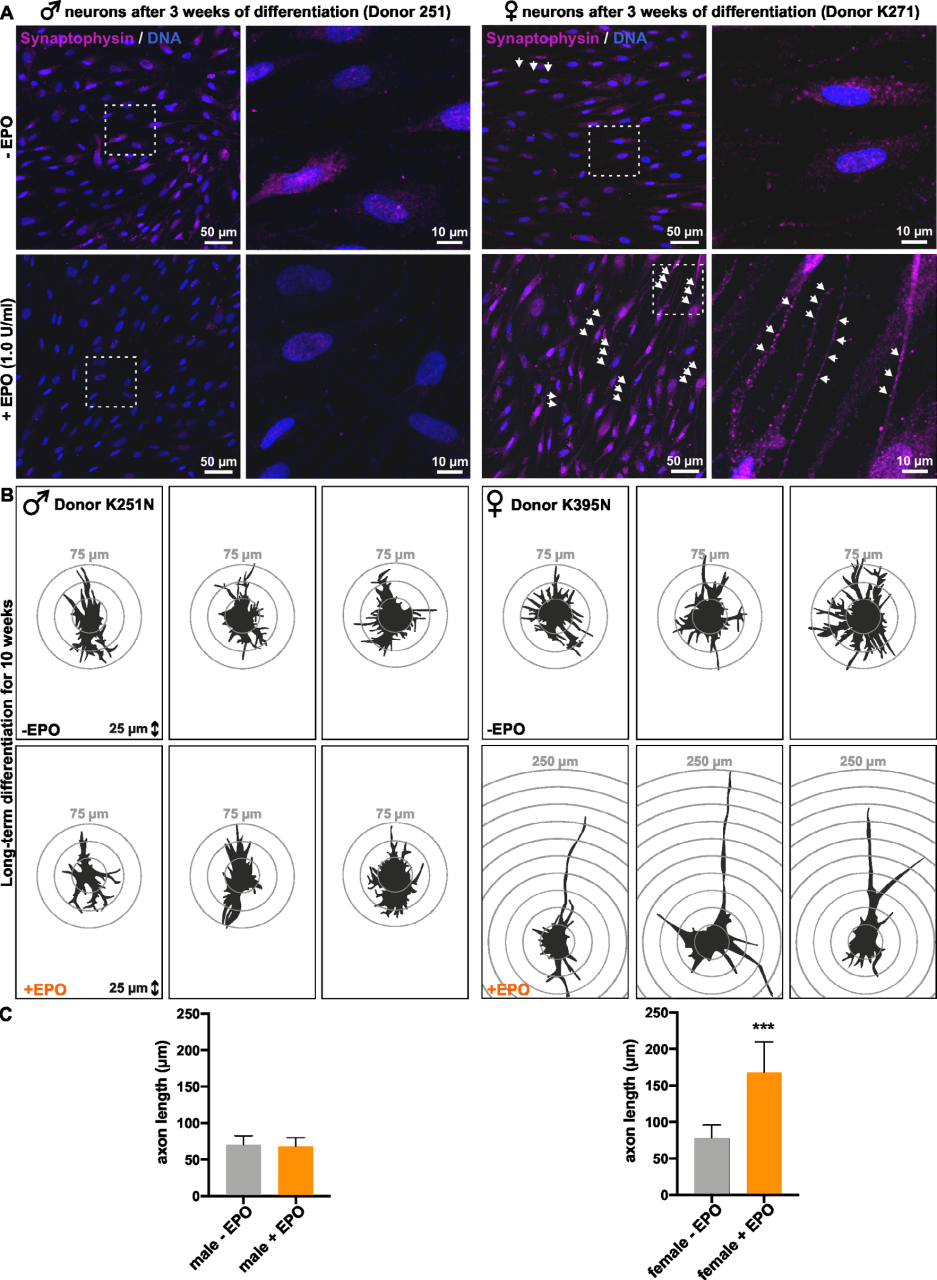
**EPO is a positive effector of neuronal differentiation in female NCSCs.** A: Immunocytochemistry revealed a slightly reduced expression of synaptophysin in male NCSCs after 3 weeks of neuronal differentiation supplemented with 1 U/ml EPO. White arrows depict synaptophysin positive presynapsis in female neurons. B, C: Camera Lucida Tracing and quantitative analysis reveals elongated axons of female NCSCs upon EPO supplementation (1 U/ml) during 10 weeks of neuronal differentiation. Mann-Whitney test, *p < 0.05, **p < 0.01, ***p < 0.001. was considered significant

To check if the enhancing effect of EPO on neuronal differentiation is also observable in male NCSCs we tested both sexes for the maturity and functionality of short-term differentiated neurons using immunocytochemistry with the neuroendocrine marker synaptophysin (Fig. 3A). Male and female NCSCs were neuronal differentiated for 3 weeks and supplemented with 1 U/ml EPO or solvent control every two days. NCSCs derived from male donors might reveal a slightly elevated expression of synaptophysin without EPO supplementation (+ EPO: 2,1% synaptophysin positive neurons, w/o EPO: 9,2% synaptophysin positive neurons) (Fig. 3A left side). Contrary, the NCSC population isolated from a female donor featured a clearly more advanced neuronal morphology. Here, especially long synaptophysin positive axons, could be observed upon EPO supplementation during neuronal differentiation (+ EPO: 91,5% synaptophysin positive neurons, w/o EPO: 15,1% synaptophysin positive neurons) (Fig. 3A, white arrows right side). In addition, we investigated whether the sex specific effect of EPO on early neuronal differentiation can also be observed in long-term differentiated NCSCs. For this purpose, we used NCSCs isolated from male and female donors for long-term neuronal differentiation (10 weeks) with EPO supplementation (1 U/ml). Camera Lucida tracing confirmed our findings, that female NCSCs enhance their neuronal differentiation in the presence of EPO (Fig. 3B right side). Especially the axonal elongation is significantly increased (p < 0,001) in female NCSCs after 10 weeks of neuronal differentiation compared to their male counterpart (p = 0,6305) (Fig. 3B, C). Female neurons supplemented with EPO show an axon length of 167,73 (SD = 41,66) µm compared to the untreated female neurons with an axon length of 77,68 (SD = 18,31) µm. In contrast, male neurons supplemented with EPO only have an average axon length of 68,37 (SD = 11,97) µm compared to the male solvent control with an axon length of 70,23 (SD = 12,89) µm (Fig. 3B, C). In summary, we provide evidence for EPO as a supportive growth factor on neuronal differentiation of female NCSCs. This applies to the expression of mature functional neuronal markers, as well as to the formation of elongated axons.

## Discussion

In this study, we reveal a novel role of the glycoprotein growth factor EPO during neuronal differentiation of human neural crest-derived stem cells. We particularly observed a strong sexual dimorphism, as the addition of EPO during neuronal differentiation increased the maturation of neurons derived from female NCSCs compared to their male counterparts.

We initially verified the expression of the EPO receptor in the NCSCs isolated from the human inferior turbinates. These inferior turbinate stem cells (ITSCs) exhibit a high differentiation potential and have already been successfully differentiated into adipocytes, neurons, osteocytes and chondrocytes [26]. Despite their origin in the embryonic peripheral nervous system (PNS), NCSCs have already been shown to be able to lose their PNS characteristics and fully differentiate into neuronal cells of the central nervous system (CNS) [35]. In addition, we previously showed, that ITSCs transplanted in a parkinsonian rat model were able to differentiate into mature dopaminergic neurons, demonstrating their ability to build functional neurons of the CNS [36].

In this line, the expression of EPOR has already been demonstrated in adult carotid body stem cells, another source for neural crest-derived stem cells [37]. Binding of EPO leads to EPOR dimerization and subsequent phosphorylation of JAK2 and activation of the EPOR. These processes results in the phosphorylation of various down-stream signaling pathways, particularly including the NF-κB signal transduction system, which is known to be involved in neuroprotection, proliferation, inflammatory response and differentiation [9, 33, 38]. In the present study, treatment of hNCSCs with EPO resulted in a profound translocation of NF-κB RELA into the nucleus. In line with our findings, EPO-mediated NF-κB activation has already been reported in differentiating rat astrocytes [39]. This fits with the results of Shingo and colleagues, who also showed nuclear translocation of RELA, but also p52 and p50 in murine neural stem cells upon EPO-treatment [40]. The importance of analyzing the specific NF-κB subunits should be emphasized, as it has already been shown that there is overexpression of the different subunits in distinct tumors [19]. In particular, overexpression of RELA is mainly found in ovarian cancer or cancer of the adrenal glands, whereas overexpression of c-REL is mainly found in lung cancer [19]. Interestingly, we very recently also observed an expression of RELA in lung cancer stem cell-like cells (LCSC-like cells), which can be stimulated by applying TNF-α [41]. Furthermore, c-REL subunit was reported to be involved in determining cell fate in adult human stem cells [21]. Additionally, the subunit heterodimers c-Rel/RELA and p50/RELA were associated with murine neural development [42]. In particular, expression of c-Rel is linked to neuronal fate, whereas inhibition of c-Rel shifts the cells into oligodendrocyte fate [21].

Since NF-κB is a key player in the development of the nervous system, we next focused on assessing potential effects of EPO-treatment on neuronal differentiation of human NCSCs. After six weeks of induced neuronal differentiation, female NCSCs revealed significantly elongated axons upon EPO supplementation. These findings extend the already described role of EPO as a regulator of differentiation of murine neural stem cells both in vitro and in vivo [40, 43]. Furthermore, Park and colleagues observed an EPO-activated effect on neuronal cell differentiation from rat neural stem cells to astrocytes [44].

EPO has also been described as a positive effector for neurodegenerative diseases in the human system. In 2013, Asadi and colleagues succeeded in significantly reducing stroke severity scores after an acute ischemic stroke by administering EPO to the patients [45, 46]. These results were also corroborated by Costa and coworkers in a study of acute spinal injury treated with EPO, which led to an improved clinical outcome of the patients [47].

As introduced above, sex is also a crucial factor for neuronal differentiation of mammalian stem cells.

In this regard, Marin-Husstege and colleagues were able to show a interaction between male and female sex hormones and the proliferation and maturation of oligodendrocyte progenitors [31]. Hillerer and coworkers also demonstrated the circulation of sex hormones to regulate hippocampal neurogenesis [32]. Various studies in the murine system have already shown that in murine system, embryonic neural stem cells display 103 differentially expressed transcripts between male and female cells in the context of proliferation and stemness states during development [48, 49]. In addition, female rats also show an increased level of granule cells and an increased number of mossy fiber synapses in the CA3 region of the hippocampus [50, 51]. In this line, neurons derived from male rats showed an increased sensibility to cell death after hypoxia exposure compared to their female counterparts [52]. Notably, sexual differences in neuronal differentiation can also be observed in the human system [53]. In this line, sex-specific differences in the course of disease and treatment efficiency can be observed, particularly in neurodegenerative diseases such as Parkinson’s disease (PD), Alzheimer’s disease and ischemic stroke [54–56]. Regarding stem cell-based neuronal differentiation, we previously discovered that female inferior turbinate stem cells significantly increased neuronal differentiation compared to their male counterparts [33]. Notably, our present findings reveal an increased presence of the mature neuronal marker synaptophysin as well as enhanced axonal length in female NCSC-derived neurons supplemented with EPO compared to their male counterparts. Interestingly, Li at al. revealed increased axonal sprouting in newborn mice cerebral cortex neurons after EPO treatment in a dose dependent manner, without examining the role of the sex [57]. In accordance to our present results, we previously demonstrated that NCSC-driven neuronal development and maturation is increased in females compared to their male counterparts [53]. Moreover, during glutamatergic differentiation of human neural crest-derived stem cells, we previously demonstrated that neurons derived from female NCSCs reveal a significantly increased RELA mediated neuroprotection against H_2_O_2_ induced apoptosis [33]. Nevertheless, the exact molecular interaction of EPO and the role of sex during neurogenesis remains ambiguous. Research into EPO mediated effects initially in vitro could be an important step in unraveling these questions.

## Conclusion

Here we demonstrate for the first time that exposure to EPO results in accelerated neuronal differentiation only in female human stem cells derived from the neural crest. Our findings add knowledge to an increasing range of studies showing sexual dimorphisms in the differentiation behavior of human stem cells [53, 58, 59]. We have analyzed sex-specific effects of Erythropoietin in human neural-crest derived stem cells during neuronal differentiation. While we detect specific morphological improvements namely axon elongation in female cell cultures only, it is currently not clear if this effect occurs also in humans. The here presented cellular system might open up a new research tools for the investigation of molecular mechanisms during sex-specific neuronal differentiation.

## Electronic supplementary material

Below is the link to the electronic supplementary material.

**Additional file 1:** **Figure S1**. EPO treatment does not result in a nuclear translocation of NF-κB RELB or c-REL. **Figure S2.** EPO treatment during one week of neuronal differentiation results in a sex-specific nuclear translocation of p65 (RELA) in female neuronal progenitors only.

**Additional file 2:** Supplementary material (original gel blot of figure 1B)

## Data Availability

All data are made available within the manuscript.
