# Supplementary material for: EPO regulates neuronal differentiation of adult human neural-crest derived stem cells in a sex-specific manner
Source: BMC Neurosci. 2023 Mar 6;24:19. doi: 10.1186/s12868-023-00789-1 (PMC9990360; doi:10.1186/s12868-023-00789-1)
Supplement: Supplementary file 1 — Additional file 1: Figure S1. EPO treatment does not result in a nuclear translocation of NF-κB RELB or c-REL. Figure S2. EPO treatment during one week of neuronal differentiation results in a sex-specific nuclear translocation of p65 (RELA) in female neuronal progenitors only. [file 12868_2023_789_MOESM1_ESM.docx]

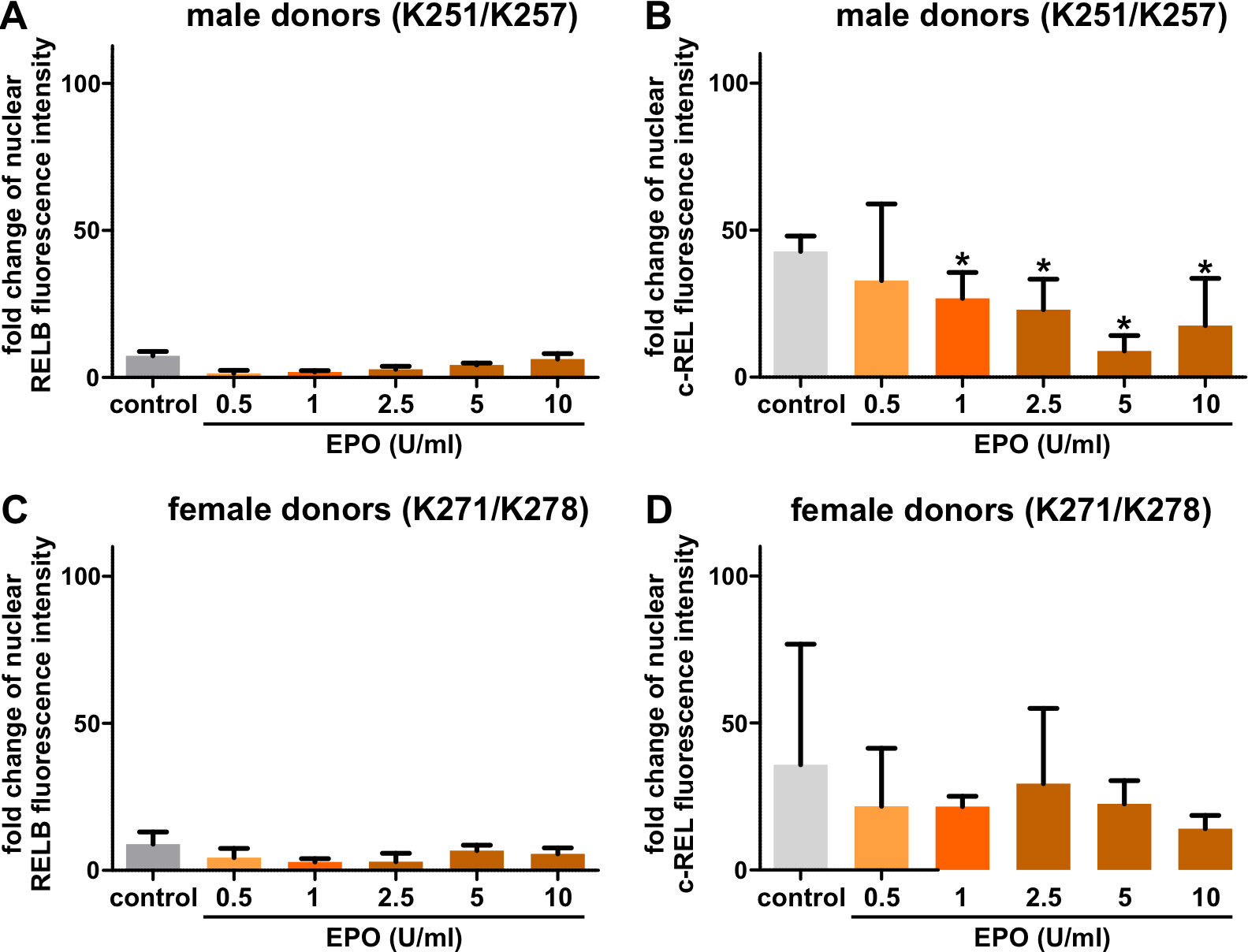


**Figure S1: EPO treatment does not result in a nuclear translocation of NF-κB RELB or c-REL** A-D: Fold change of nuclear fluorescence intensity. There was no increase of nuclear translocation of RELB or c-REL after EPO treatment in male and female NCSCs, but a slight decrease of c-REL was observed in male NCSCs. Mann-Whitney test, *p<0.05, **p<0.01, ***p<0.001. was considered significant.


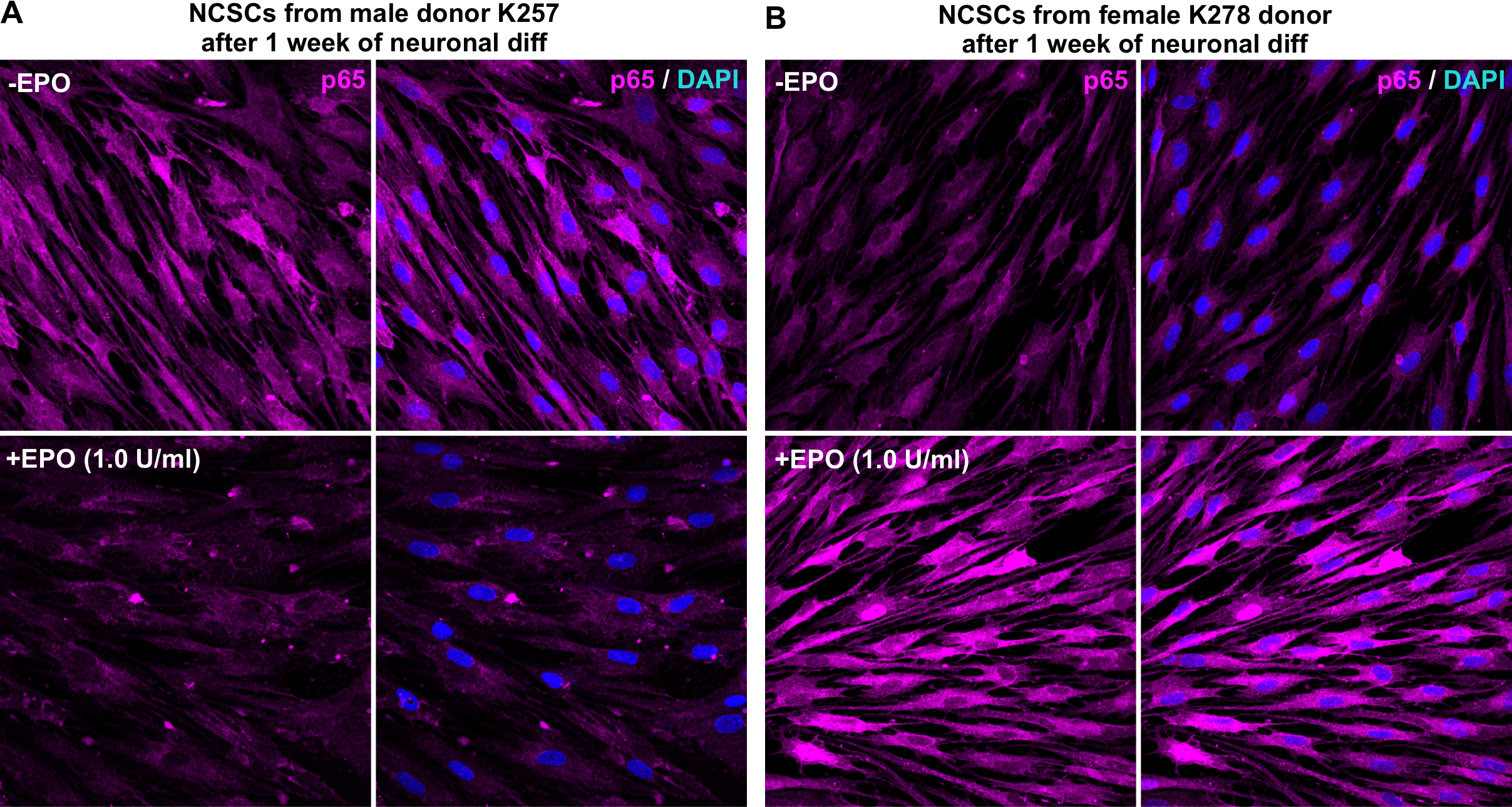


**Figure S2: EPO treatment during one week of neuronal differentiation results in a sex-specific nuclear translocation of p65 (RELA) in female neuronal progenitors only.** A: One week of neuronal differentiation with 1 U/ml EPO supplementation results in a reduced nuclear translocation of NF-κB p65 in neurons derived from male ITSCs. B: One week of neuronal differentiation with 1 U/ml EPO treatment showed an increased nuclear translocation of NF-κB p65 in neurons derived from female ITSCs.
