# Supplementary figures and images for: EPO regulates neuronal differentiation of adult human neural-crest derived stem cells in a sex-specific manner
Source: BMC Neurosci. 2023 Mar 6;24:19. doi: 10.1186/s12868-023-00789-1 (PMC9990360; doi:10.1186/s12868-023-00789-1)

Supplementary Information:

Figure 1B)


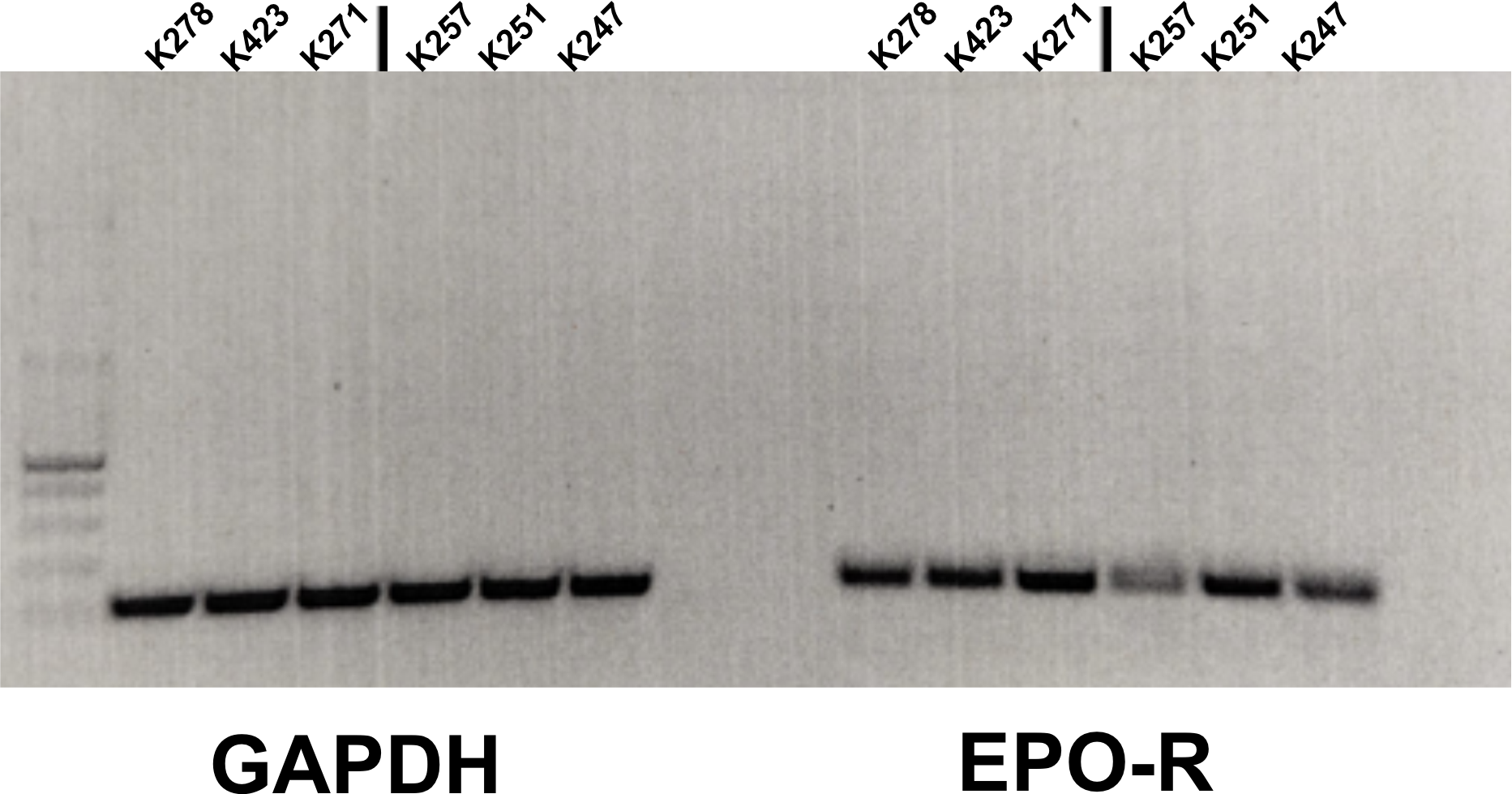

Supplement: Supplementary file 2 — Additional file 2: Supplementary material (original gel blot of figure 1B) [file 12868_2023_789_MOESM2_ESM.docx]
